# Acute nicotine poisoning in a 2-year-old child - A case report of a near-death event

**DOI:** 10.51866/cr.532

**Published:** 2025-10-18

**Authors:** Mohd Shaiful Ehsan Shalihin, Sakinah Md Rifin, Nur Izzati Binti Mohd Sabri

**Affiliations:** 1 MBBS, MMED FAM MED, Department of Family Medicine, Kulliyyah of Medicine, International Islamic University of Malaysia, Jalan Sultan Ahmad Shah, Kuantan, Pahang, Malaysia. E-mail: shaifulehsan@iium.edu.my; 2 MBBS, MMED FAM MED, Klinik Kesihatan Bandar 32 Bera, Jalan Tementi, Sebertak Bera, Pahang, Malaysia.; 3 MD, Klinik Kesihatan Bandar 32 Bera, Jalan Tementi, Sebertak Bera, Pahang, Malaysia.

**Keywords:** Poisoning, Child, Nicotine, Respiratory insufficiency

## Abstract

Acute nicotine poisoning is a devastating condition that occurs when there is an excessive intake of nicotine, a toxic alkaloid found in tobacco products, mainly e-cigarettes. It impairs respiratory and neurological functioning due to its massive inflammatory effect. It is mainly related to vaping activity. Most smokers know about smoking’s adverse effects and implications. However, public awareness is still poor, and a majority are unable to quit the trending vaping addiction. Nevertheless, the potential severity of its toxicity to a growing child both in the short and long term is of critical concern, especially in cases of accidental direct ingestion. We report the case of a 2-year-old child who presented with acute respiratory failure secondary to liquid nicotine ingestion at home. The child was first seen by the medical team at primary care and required urgent intubation and paediatric intensive care unit admission. Her condition was complicated with several episodes of seizure requiring close monitoring. This case highlights that despite ignorance of adults on the danger of vaping, significant harm can still occur among children at home when parents engage in vaping. Although adults may take certain precautions, their harmful habits can indirectly and directly affect children, making the risk unavoidable. This serves as an urgent call for the government to strictly ban nicotine products and enact corresponding legislation immediately.

## Introduction

Electronic cigarettes (e-cigarettes) are popular nicotine delivery products highly used by the public. However, e-cigarettes lack an established safety profile due to inadequate studies on their short- or long-term effects on users.^1^ Their effects are not well-explained even in infants and children. Nevertheless, cases of lung injury have been reported as early as adolescence.^2^ Liquid nicotine could also be accidentally ingested or inhaled by children. Parents’ lack of awareness on nicotine effects may be main contributors to potentially devastating sequelae on growing children.

E-cigarettes can induce adverse respiratory and cardiovascular effects through an inflammatory process, accumulation of oxidative stress, DNA alteration, platelet disturbance and arterial stiffness. Their use can triple the risk of developing myocardial infarction and cause respiratory distress even in healthy individuals,^3^ therefore potentially leading to sudden death. Children of smokers are directly exposed to nicotine and other harmful tobacco smoke chemicals starting in utero and continuing in the home environment. These effects place children at an increased risk of adverse health outcomes later in life, such as obesity, behavioural problems and cardiovascular disease.^3^

Liquid nicotine in e-cigarettes can be easily ingested or inhaled by children if not supervised accordingly. Even e-cigarettes can be misinterpreted by children as toys. Children often mimic the behaviour of their parents, a tendency typically occurring among those aged 1-2 years.^4^ The colourful packaging and uniquely shaped design of e-cigarettes can appear visually appealing to young children. Parents may unintentionally leave e-cigarettes on tables, sometimes unattended. In the worst-case scenario, a child may be able to reach the device, leading to direct exposure. These situations can lead to accidental acute nicotine ingestion.

## Case presentation

A 2-year-old girl was brought in by her parents to a primary care clinic with complaints of sudden breathlessness and reduced consciousness lasting 5 min, which were preceded by an accidental ingestion of liquid nicotine at home about 15 min from the arrival. The incident was discovered by the child’s sister at a time when the parents were not present with the children. The child had no other associated symptoms, except for one episode of vomiting after the ingestion. The vape liquid was pink and had a strawberry flavour.

Upon arrival, the child was unconscious with a GCS score of 3/15 (E1, V1, M1). She had no spontaneous breathing. No heart sound was heard, and her pulses were not palpable. Cardiopulmonary resuscitation commenced immediately for 2 min, resulting in the return of spontaneous circulation. Intubation was conducted, and the airway was protected, with her oxygen saturation maintained at 99%. Her heart rate was 120 bpm. Without further delay, she was transferred to a tertiary hospital and required close monitoring in the paediatric intensive care unit. The health district office was contacted, and the possibility of acute e-cigarette or vaping use-associated lung injury (EVALI) was reported. The social worker and child protector were informed about the incident to rule out any possibility of parental negligence.

During admission, the child experienced two episodes of seizure and required an intravenous infusion of phenytoin. She was extubated after 4 days on ventilatory support. Urine investigations revealed the presence of cotinine, a nicotine metabolite. Both her chest radiograph and brain CT scan showed normal findings, and other investigations also yielded unremarkable findings. Upon transfer to the general ward, the child exhibited difficulty walking and talking. An MRI of the brain was performed, which revealed normal findings. She benefitted from combined care, including follow-ups with a physiotherapist, occupational therapist and speech therapist. Her condition gradually improved, and she was discharged in stable condition after nearly 2 weeks of hospitalisation.

The child’s growth and developmental milestones were closely monitored by a family medicine specialist at the primary care clinic. Upon further exploration, both parents were found to be supportive and actively involved in their children’s care. They had neither financial difficulties nor marital issues. Home visit reports and feedback from social workers revealed no evidence of child abuse or neglect. Following the incident, the father was able to quit his nicotine addiction with support from the quit-smoking clinic, highlighting a positive outcome of the event.

## Discussion

Acute nicotine poisoning is a growing concern, particularly among children. The increasing use of nicotine-containing products, such as e-cigarettes, has amplified the risk of exposure. These products often contain concentrated liquid nicotine, which can lead to severe toxicity if ingested, inhaled or absorbed through the skin.^5^ The prevalence of acute nicotine poisoning in children has risen significantly in recent years, in view of the growing popularity of vaping among the public over the past decade.^6,7^ More than 8000 cases of nicotine exposure had been reported in children younger than 6 years from 2012 to 2017, averaging 129 cases per month worldwide. As in our case, the ingestion of liquid nicotine accounted for most incidents, particularly in children under 3 years old, due to accidental exposure to e-cigarette refills.^7^ In Malaysia, the first case of nicotine poisoning among children was reported in 2021, where a 9-year-old boy who vaped in secret for 4 days in a row using a family member’s disposable pod vape fell unconscious at his house after experiencing vomiting and dizziness.^8^ Notably, a mortality case was reported in a 16-year-old child earlier this year due to acute heart failure and pulmonary embolism after a 3-year history of vaping.^9^

The role of e-cigarette use in smoking cessation is limited and unapproved in scientific research.^10^ However, the public is still unaware of its similar or even higher hazard risk to users in comparison to conventional cigarette use. Furthermore, it is difficult to determine the contents and concentration of nicotine in e-cigarettes.^10^ Some individuals may modify the products or add other substances, such as stimulants and opioids, to enhance their enjoyment and leisure satisfaction, which may lead to drug addiction and abuse.^11^ Therefore, the potential implications and consequences of e-cigarette access among children, especially preschoolers and infants, are deeply concerning. In our case, the child was as young as 2 years old, with effects that were both severe and unpredictable. Substances found in e-cigarettes are dangerous to health. Propylene glycol, glycerine and carbonyl compounds are humectants and carcinogenic substances that generate pulmonary irritants. Metals present in heating coils and cartridge casings, especially aluminium, chromium, iron, lead, manganese, nickel and tin, may leach during use.

In this report, the vape liquid was pink, with an added strawberry flavour and smell, which appeared highly appealing to the child. The addition of other substances may further amplify the nicotine effects. Furthermore, flavouring agents contained within e-cigarette cartridges lack safety data and have been associated with the development of bronchiolitis obliterans.^10^ Vitamin E acetate has also been detected in e-cigarettes; as it cannot be metabolised by the body, it may accumulate and lead to toxicity.^12^ Acute nicotine poisoning, even from small amounts (1 mg), can lead to overstimulation of nicotinic receptors, resulting in nausea, vomiting, increased salivation, abdominal pain, diarrhoea, tachycardia and hypertension.^13^ This is followed by a later phase of receptor desensitisation and paralysis, particularly hypotension, bradycardia, muscle weakness, confusion, seizures and coma.^13^ In this case report, the patient presented with symptoms suggesting the involvement of multiple organs, specifically the respiratory, gastrointestinal and central nervous systems. She was in acute respiratory distress preceding episodes of vomiting, which was later followed by an episode of seizure in the ward. Had the parents delayed in seeking medical attention and consequently in stabilising the child, the outcome could have been fatal. Even though the child survives, the nicotine effects could be permanent, especially in terms of damage to the developing brain, and may lead to unfavourable long-term sequelae affecting the child’s future health.^14^

Children can readily acquire affordable vapes and e-cigarettes since merchants do not inquire about buyers’ age at the time of purchase.^14^ Children whose parents are vapers are also more likely to engage in vaping.^15^ This is likely because children view their parents as role models from a young age, and parental habits are easily mimicked, especially if parents do not consider their own actions as harmful. Furthermore, the bright colours and fancy packaging of e-cigarettes appeal to children, encouraging them to try and use these products.^16^ Consequently, children are more likely to handle and experiment with e-cigarettes when devices are readily accessible, regardless of their parents’ knowledge. Thus, laws and legislation are urgently needed to address this issue. The government’s move to prevent tobacco product use among future generations through the implementation of Act 852: The Control of Cigarette Products Act should be supported by all sectors and the public.^17^ Its enforcement aims to create a new generation free from any tobacco products. Parental awareness regarding the effects of vaping on children and the risk of encouraging future vaping habits remains low.^18^ Some parents do not express concern if their children engage in vaping and fail to discuss its negative effects openly.^19^ An analysis has shown a favourable relationship between parental knowledge and how children and adolescents perceive the harm and prevalence of vaping.^16^ Even if parents are unable to quit vaping, e-cigarettes should not be left accessible at home, and strict household rules should be enforced to prevent children from seeing or accessing these devices.^20^

Active measures should be initiated by parents immediately to prevent nicotine exposure among their children. Parents should ensure their children are not exposed to second-hand emissions from any tobacco products, including e-cigarettes, by abstaining from tobacco use themselves and thereby setting a positive example. Indeed, parents should prioritise quitting smoking.^21^ They should also actively educate their children or teenagers about the dangers of e-cigarettes, as there is always time for open discussion. Additionally, parents should clearly communicate that they do not want any family members to use tobacco products, including e-cigarettes, as they are harmful. If parents require assistance, they should seek support from community clinics, support groups and non-governmental associations.

## Conclusion

Nicotine ingestion in children is a medical emergency that can result in mortality if not managed promptly. The effects are particularly severe in young children and may lead to detrimental long-term sequelae. Although early airway and circulation support can improve outcomes, the best approach is prevention. This can only be achieved if adults refrain from smoking in the first place.
